# Learning on Jupiter, learning on the Moon: the dark side of the G-force. Effects of gravity changes on neurovascular unit and modulation of learning and memory

**DOI:** 10.3389/fnbeh.2012.00064

**Published:** 2012-09-24

**Authors:** Yves Porte, Jean-Luc Morel

**Affiliations:** ^1^Université de BordeauxBordeaux, France; ^2^Centre National de la Recherche Scientifique Unité Mixte de Recherche 5293, Institut des Maladies NeurodégénérativesTalence, France

**Keywords:** learning and memory, cognition, emotion, gravity, hypergravity, microgravity, stress

## Abstract

On earth, gravity vector conditions the development of all living beings by physically imposing an axis along which to build their organism. Thus, during their whole life, they have to fight against this force not only to maintain their architectural organization but also to coordinate the communication between organs and keep their physiology in a balanced steady-state. In space, astronauts show physiological, psychological, and cognitive deregulations, ranging from bone decalcification or decrease of musculature, to depressive-like disorders, and spatial disorientation. Nonetheless, they are confronted to a great amount of physical changes in their environment such as solar radiations, loss of light-dark cycle, lack of spatial landmarks, confinement, and obviously a dramatic decrease of gravity force. It is thus very hard to selectively discriminate the strict role of gravity level alterations on physiological, and particularly cerebral, dysfunction. To this purpose, it is important to design autonomous models and apparatuses for behavioral phenotyping utilizable under modified gravity environments. Our team actually aims at working on this area of research.

## Introduction

Why studying the influence of gravity on living organisms since it operates continuously in all living entities on Earth? Obviously this question is crucial for space exploration, but it also brings with it an aspect of basic research to understand how gravity has participated in shaping the living. The development of life on Earth integrates 1 G gravity vector value. In plants, it has been determined that root growth is sensitive to gravity changes in a 10^−3^–10^−4^ G range (Driss-Ecole et al., 2008). To investigate this issue in mammals there are two possible ways: either to reduce (microgravity) or increase (hypergravity) the level of gravity. Microgravity can be studied by placing the animal in space or in another planetary environment (for instance, 0.16 G on the Moon), or by simulation on Earth by electromagnetic levitation. On the contrary, hypergravity can be experienced on very massive planets like Jupiter (2.35G) or simulated by centrifugation on Earth. In this research framework, study of the effects of gravity changes is particularly interesting since it is a physical environmental change applicable on the whole animal (*in vivo*) as on cells or tissues (*ex vivo*), thereby separating direct effects (gravity-induced cellular modifications) from physiological adaptation (hormonal and/or nerve regulations). The major sources of problems with studying real gravity changes as evoked by other planetary environments (Moon, Mars, …) is that it also modifies the nature and levels of radiations, and is very expensive. On the contrary, hypergravity developed in centrifuge is easy to implement (instrument, intensity, duration, developmental stages…) and almost exclusively affects the gravity variable. But once the centrifuge built, it remains that instrumental development automation is needed to measure parameters *in vivo* in a basket centrifuge. Once elaborated, these automated processes can be exported to conventional laboratory with the major advantage that they drastically reduce contacts between animals (experimental subjects) and human (experimenter).

Three years ago, we have organized a consortium of laboratories to study how an increase of gravity vector affects the physiological functions in mice. This project was supported by grants from the ANR and equipment built by CNES. The studies that we have made indicated that many functions were affected after 21 days exposure to hypergravity (vestibular reflexes, muscle force and phenotype, bone architecture, vessel activity, immune response, circulating hormones, locomotion, etc.). Our expertise in the “gravity effect” studies was initially in vascular field and our efforts have focused on cerebral vascular function. Indeed, we aim at working on the role of cerebral vascular function in memory processes and more specifically how the neurobiological processes involved in memory could be affected by changes or adaptations of the cerebral vasculature functions.

As we expose in this review, the effects of gravity changes were measured after a period of exposure to another level of gravity. The proposed cognitive effects rely then on a series of *a posteriori* taken images of the situation, measured globally on a group of animals. However, our work and probably others in this field of investigation indicated that the individual response, and the time course of behavioral adaptation of each subject, should be examined to be correlated to individual molecular studies.

## Gravity, blood flow, and cerebral function

Among all environmental parameters that can alter cerebrovascular reactivity, variations in the level of gravity have been described as a candidate by re-equilibrating blood perfusion. Indeed, in human, reduced gravity such as that experienced in space, induces corporal fluids' re-distribution leading to the loss of head-to-foot hydrostatic pressure gradient (Convertino et al., 1989; Norsk, 1992; De Santo et al., 2001). However, this is less evident in animal models such as rodents because of their quadruped station which obviously reduces the initial head-to-foot pressure gradient. The resulting effect of weightlessness achieved in spaceflights is a highly complex vascular adaptation to the increase in cardiac output by reducing the systemic vascular resistance, which limits the increase of blood pressure (reviewed in Norsk and Christensen, 2009). It is suggested that the opposite effect is observed in cerebral arteries. The gravity changes can therefore induce a vascular adaptation to counteract any modification of cerebral perfusion.

Vascular dysfunctions are also described as risk factor or associated symptoms in several neurodegenerative diseases. Classically, ischemic stroke, atherosclerosis, hypertension, and cardiac disease have been reported to result in cerebrovascular disease and potentially trigger Alzheimer disease in older adults (de la Torre, 2009; Viswanathan et al., 2009; Austin et al., 2011; Mazza et al., 2011). Orthostatic hypotension, the result of the autonomic perturbation observed in astronauts after spaceflight, is also described as a complication or symptom in 18–81% of the Parkinsonian patients (Ha et al., 2011). Another concomitant non-motor complication of Parkinson disease is the cognitive impairment and both are not stemmed with drug treatments reviewed by Lyons and Pahwa (2011) and Jain and Goldstein (2012). Thus, at this stage of our proposal, it is possible to link, on the one hand, cognitive impairments with modification of brain perfusion due to vascular dysfunction, and on the other hand the gravity changes and modifications of vascular function. Indeed, gravity changes could alter cognitive function via modulation of vascular reactivity.

In the brain, neuronal metabolism almost essentially implies glucose oxidation. Then, in all animal species, brain well-functioning closely depends on oxygen availability. Oxygen is brought to neurons by cerebral blood flow, which is at least in part, regulated by vascular smooth muscle cells (VSMCs) contractility/reactivity and depends on intracellular calcium concentrations. These last are regulated by the activity of three major classes of actors in the cells: (1) calcium entry through voltage- or non-voltage-dependent calcium channels at the plasma membrane, which initiates (2) calcium-dependent calcium release from endo-sarcoplasmic reticulum through inositol-1,4,5-trisphosphate receptors (InsP3R) and/or ryanodine receptors (RyRs), stopped thanks to (3) calcium stores refilling (Sarco/Endoplasmic Reticulum ATPase pumps, SERCA) or calcium extrusion (Plasma Membrane Calcium ATPase, PMCA). However, these phenomena are under the control of another calcium-signaling actor family composed of channels and regulators implicated in calcium entry and control of calcium stores refilling (STIM/ORAI/CRACR2 and SARAF).

Calcium signals regulate VSMCs reactivity. Briefly, vasoconstriction is due to propagated calcium waves encoded by InsP3R activation via G-Protein Coupled Receptor (GPCR)/PLCβ pathways or by RyR opening via calcium entry after depolarization (CaV channels) or cyclic-ADP ribose pathways activation reviewed in Morel et al. (2007) and Berridge (2008). But calcium signals also regulate vasorelaxation through localized and brief calcium signals named calcium sparks encoded by RyR and leading to the increase of activity of calcium-activated potassium channels named BKCa (Nelson et al., 1995). Thus, as all excitable cells, the VSMCs also express many other ionic channels as potassium channels (Kitazono et al., 1995) that are able to modulate membrane potential to regulate their level of contractility.

In fact, VSMCs reactivity can be modulated by neurons, either directly or via astrocytes, to adapt cerebral blood flow to cell needs, as well as by the endothelium to adapt vessels function to the blood pressure and cardiac output. As reviewed by Attwell et al. (2010) the neuronal and glial control of brain blood flow is essential for oxygen and glucose inputs.

**Figure 1:**
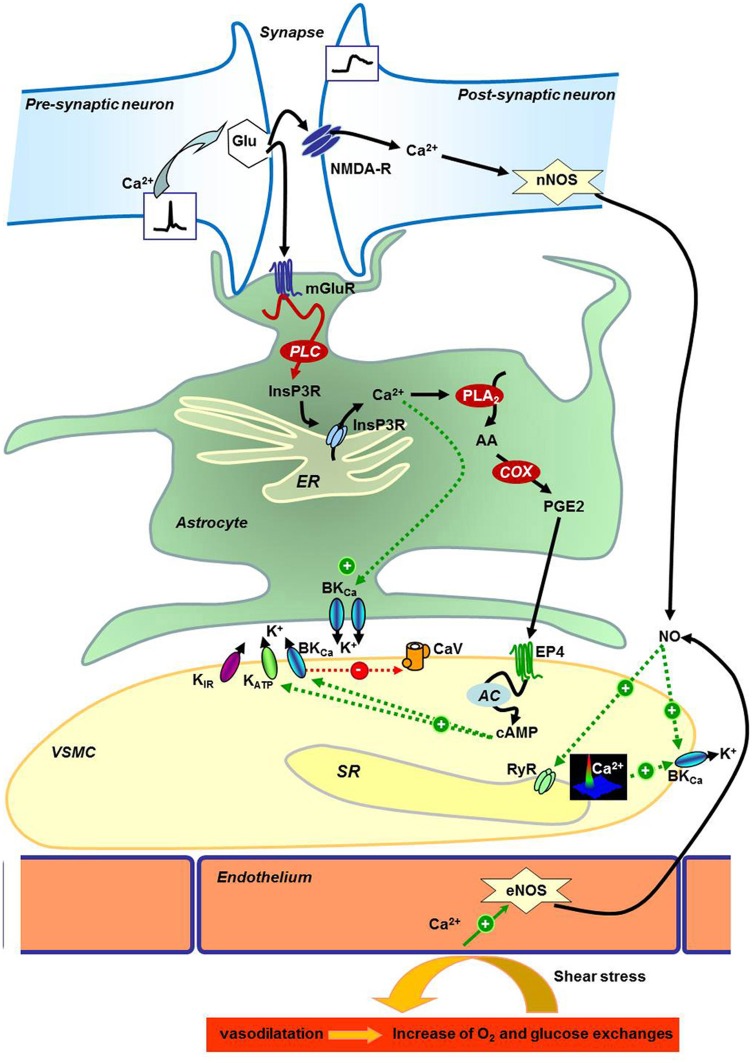
**Neurogliovascular control of cerebral perfusion.** Schematic representation of neuronal, astrocyte, and endothelial molecular control of vascular smooth muscle cells reactivity in the brain. AA, Arachidonic acid; AC, Adenylyl Cyclase; BK_Ca_, Calcium-activated potassium channels; Ca^2+^, Calcium; cAMP, cyclic Adenosin Monophosphate; CaV, Voltage gated Calcium channel; COX, Cytochrome c Oxydase; eNOS, Endothelial Nitric Oxide Synthase; EP4, Prostaglandin E Receptor 4; ER, Endoplasmic Reticulum; Glu, Glutamate; InsP3R, Inositol-1,4,5-trisphosphate Receptor; K^+^, Potassium; K_ATP_, ATP-dependent Potassium channel; K_IR_, Inward rectifier Potassium channel; mGluR, Glutamate metabotropic receptor; NMDA-R, N-Methyl-D-Aspartate Receptor; nNOS, Neuronal Nitric Oxide Synthase; NO, Nitric Oxide; O_2_, Dioxygen; PGE2, Prostaglandin E2; PLA_2_, Phospholipase A_2_; PLC, Phospholipase C; RyR, Ryanodine Receptor; SR, Sarcoplasmic Reticulum; VSMC, Vascular Smooth Muscle Cell.

In Figure 1, we tried to summarize these neuroglial pathways: (1) in neurons, the presynaptic release of glutamate activates the post-synaptic NMDA receptor to encode calcium signal inducing activation of the neuronal nitric oxide (NO) synthase (nNOS) and vasodilatation (Domoki et al., 2002; Zonta et al., 2003; Busija et al., 2007). The released NO can then modulate activity of RyR and calcium-activated potassium channels (BKCa) to hyperpolarize VSMCs and dilate cerebral artery (Mandala et al., 2007; Yuill et al., 2010); (2) glutamate also binds metabotropic receptors on the astrocyte membrane to activate a calcium wave (Filosa et al., 2004) and cytochrome C oxydase (COX) to produce prostaglandin PGE2 and epoxyeicosatrienoic acid EET (Zonta et al., 2003) responsible for vasodilatation via the increase of potassium channels activity (Filosa et al., 2006). As summarized in Dunn and Nelson (2010), the EET are produced by the action of cytochrome P450 epoxygenase CYP4A on 20-hydroxy-eicosatetraenoic acid (20-HETE). This enzymatic reaction increases vasodilatation by the produced EET known to increase BKCa activity directly or via the increase of calcium spark frequency; and also decreases the 20-HETE concentration (20-HETE is described to potentiate vasoconstriction via the inhibition of BKCa). The stimulation of potassium channels induces hyperpolarization and decreases the CaV activity. Other potassium channels expressed in VSMC are also implicated: inward rectifier potassium channels (K_IR_) are activated by the increase of extracellular potassium concentration due to astrocyte's BKCa activation, and ATP-activated potassium channel (K_ATP_) activity is increased by phosphorylation by cAMP-dependent kinase PKA. Resulting vasodilation is necessary for the increase of dioxygen (O_2_) and glucose availability for neurons, a cause of functional hyperemia. The opposite reaction is in part due to the VSMC contraction to decrease the exchange between blood and neurons. Vasoconstriction can be produced not only via GPCR activation by hormones like angiotensin-II evoking calcium waves (reviewed in Morel et al., 2007), but also by 20-HETE derived from arachidonic acid (AA) that inhibits BKCa to depolarize the VSMC plasma membrane and consequently increases the calcium entry by CaV. The most studied pathways (PGE2, EET, and NO production) are sensitive to O_2_ concentrations at different levels, and then O_2_ can regulate vasodilatation and functional hyperemia by itself. But it is not excluded that other mechanisms may be activated to maintain or disrupt vasodilation and/or glucose and O_2_ transports. Thus, calcium signaling is a crucial step in the message transduction between cells in the neurogliovascular unit and for the regulation of VSMC contractile status. For these reasons, exploration of the calcium signals, in VSMC from animal models submitted to gravity changes, have been performed.

In this context, we recently demonstrated that spaceflight regulates portal vein myocytes calcium signaling in the opposite way of hypertension. More precisely, mice exposed to microgravity during an eight-day shuttle flight, as well as hindlimb unloaded rats, displayed decreased expression of RyR 1 expression in VSMCs from hepatic portal vein, associated with decreased calcium-induced calcium release signals. We demonstrated that these cells are *per se* directly sensitive to microgravity and adapt their intracellular signaling even in culture preparations. In this study, we have shown for the first time that real and simulated microgravity applied on animals and cultured cells have similar effect in terms of gene expression. Interestingly, in spontaneously hypertensive rat's portal vein VSMCs, RyR 1 expression, and associated calcium signals were increased (Dabertrand et al., 2012). Taken together, these recent data suggest that microgravity can effectively be modeled in rodents by caudal suspension, and acts in the same way as in human by decreasing peripheral blood vessels pressure when increasing cerebral arterial one.

When considering the hypergravity side of the problem, we are unfortunately forced to note that the effects of higher gravity levels on blood pressure are not well known. However, one can reasonably suppose that hypergravity can modify cerebral blood flow too. Our work in this field of investigation brought several lines of evidence supporting this hypothesis. For instance, and to respond to our previous works on microgravity, we recently investigated cerebral arteries VSMCs calcium signaling in adult male mice bred under hypergravity conditions (3G) during 21 days. If the breeding of animals in hypergravity is easier than raising a space module, the fact remains that the investigative methods must adapt to the small size of the samples and the large number of target to study. For example, RT-qPCR experiments, associated with western-blots and immunolabeling in mice may allow understanding how the expression of different pumps and channels may be affected by hypergravity. Then, it is noteworthy that gravity, as a physical constraint for the organism, can at least modulate cerebral, and thus cognitive, functions. However, studies in humans and rodents have only recently targeted learning and memory alterations in the field of altered gravity physiological effects, during development as well as at the adult stage (Sajdel-Sulkowska, 2008; Zago et al., 2009).

## Blood brain barrier (BBB), cerebral function, and gravity

Although cerebral blood flow is essential for the well-functioning of the brain, blood system not only conveys oxygen and nutriments, but can transport toxins, bacteria, viruses, chemicals, or drugs too, each of them being potentially able to severely alter the integrity of neurons. To fight against these potential aggressors, a physical barrier exists between blood and neuronal compartments, the blood brain barrier (BBB), which acts as a semi-permeable filter. The integrity of the BBB especially depends on tight junctions between pericytes and endothelial cells, as demonstrated recently in pericytes deficient mice (Bell et al., 2010). In 1970's, Rapoport suggested that acute hypertension induces a degradation of BBB (Rapoport, 1976). This hypothesis was effectively evidenced almost thirty years later by extravasation of IgG (Kuang et al., 2004) and increase of reactive oxygen species in the cortex and the hippocampus (Poulet et al., 2006). In chronic hypertensive model, like spontaneous hypertensive rats (SHR) or stroke-prone hypertensive model (SPSHR), the leak of serum proteins through BBB was observed in the hypothalamus (Ueno et al., 2004), as well as extravasation (Al-Sarraf and Philip, 2003), associated with an increase of expression of P-glycoprotein in vessels (Ueno et al., 2009).

On the other hand, molecular processes regulating the neuro-glio-vascular unit activity are implicated in BBB modifications. For example, the stimulation of NO-pathway decreases the effect of acute hypertension on BBB (Mayhan, 1995). Moreover, calcium signaling regulates tight junctions, as indirectly illustrated by the beneficial effects of dihydropyridine or PKC inhibitor treatments on hypertension related BBB damages (Turkel et al., 2004; Qi et al., 2008). In the same order of idea, antihypertensive AT1-R antagonists reduce the BBB alterations caused by acute hypertension in diabetic rat (Kaya et al., 2003). Indeed, a lot of arguments can be exposed in favor of a link between cerebral blood pressure and BBB integrity. But this link goes further when considering the consecutive effects on cerebral function. For example, in neurodegenerative diseases, alterations of BBB have been described as potentially increasing symptoms. Especially in Alzheimer's disease, Zlokovic group defends the idea that cerebrovascular dysregulation is an important feature participating to the cognitive decline (Zlokovic et al., 2005). In particular, brain vessels would be implicated in amyloid peptide clearance in normal brain, a function that could be dysregulated in Alzheimer's patients (Sagare et al., 2012). Recently, it was demonstrated that the surgical induction of hypertension by transverse aortic coarctation was able to produce learning and memory impairments in Morris water maze and novel object recognition task, associated with brain accumulation of amyloid peptide (Carnevale et al., 2012). This can be related with BBB damages as the DHP treatment able to decrease BBB alterations is also capable to increase the amyoloid peptide clearance (Paris et al., 2011).

Thus, as hypertension seems to be closely related with BBB alterations (and consecutive cognitive impairments), it is easy to speculate that microgravity effects on learning and memory depend on BBB opening. If the visible oedema (puffy face) and the effective measurement of cerebral hypertension induced by microgravity or hindlimb unloading are in line with this eventuality (Lakin et al., 2007), to our knowledge, very few studies were interested in this research field. In hindlimb unloaded rabbits, the integrity of BBB does not seem to be drastically affected (Shimoyama et al., 2000). On the contrary, in rat, three exposures to lower body negative pressure induced increasing of brain water content and lanthanum extravasation (Sun et al., 1997). In area postrema, Virchow-Robin spaces were modified suggesting a modification of BBB (Pashchenko and Sukhoterin, 2000). But the most described phenomenon is the modification of choroid plexus. In the first 30 min after hindlimb suspension, the proteins implicated in cerebrospinal fluid secretion are more expressed whereas Aquaporin-1 expression is decreased (Masseguin et al., 2000, 2001). After adaptation these parameters return to normal (Masseguin et al., 2001) or are regulated in the opposite way (Masseguin et al., 2000). Taken together, these results indicate that the very beginning of unloading (suspension or microgravity exposure) is a critical period in which BBB alterations can occur. Among the possible causes, the interaction between confinement and elevated temperature observed during space flight can be cited. In fact, BBB damages could be observed in rat placed 4 h at 38°C and this effect was potentiated in hypertensive rat (Muresanu et al., 2010). The chronic diet risk factors (cholesterol, ethanol) also induced BBB damages in rats probably by increasing inflammation and amyloidosis (Ehrlich and Humpel, 2012), and this is not trivial when considering the diet of astronauts…

## Cerebrovascular network, memory, and gravity

If neurogenesis does not really appear to be essential for enhancing learning and memory (Shors et al., 2002; Meshi et al., 2006), angiogenesis in cerebral structures implicated in memory, such as the hippocampus, could be critical. Indeed, a recent pharmacological study in Morris water maze showed that blocking neurogenesis failed to alter acquisition and long term retention when blocking angiogenesis did (Kerr et al., 2010). Despite the fact that pro-angiogenic molecules such as vascular endothelial growth factor (VEGF) have several effects on neurons (for review, Mackenzie and Ruhrberg, 2012) they can ameliorate memory via angiogenesis in transgenic model of Alzheimer Disease (Wang et al., 2011). Moreover, data have been suggesting that complex environment and social interactions increased dendrites in neurons and also the microvessels network in cortex (Black et al., 1987; Sirevaag et al., 1988; Wallace et al., 2011). In this case, the time course of the capillary sprouting appeared after dendrites one (Wallace et al., 2011).

In parallel, vessels were shown to be sensitive to gravity: *in vitro* studies indicated that microgravity increased or decreased the proliferation of endothelial cells from large and micro vessels respectively. When the proliferation was increased, the expressions of Hsp70 and Il-6 were decreased and p21, NOS, and MPC-1 were increased (Carlsson et al., 2003; Cotrupi et al., 2005). Thus, it is obvious to speculate that the cognitive and cerebrovascular statuses are nested. Life in other gravity context implicates modifications of social interactions, impoverishment of context stimulations, another management of exercise, increase of chronic and acute stress, and of course a change in gravity level. This is why we propose now to understand how all of these parameters can interact by studying (*ex vivo* and *in vivo*) vascular (integrity of BBB, vasoreactivity, neuro-astro-pericytes interactions) and neuronal (electrophysiological, cognitive levels) networks in animals living in modified gravity. This analysis should then take into account various other environmental parameters such as with different levels of social interactions, context stimulation, exercise, stress, and diet.

## Gravity, mood, and affect

**Figure 2:**
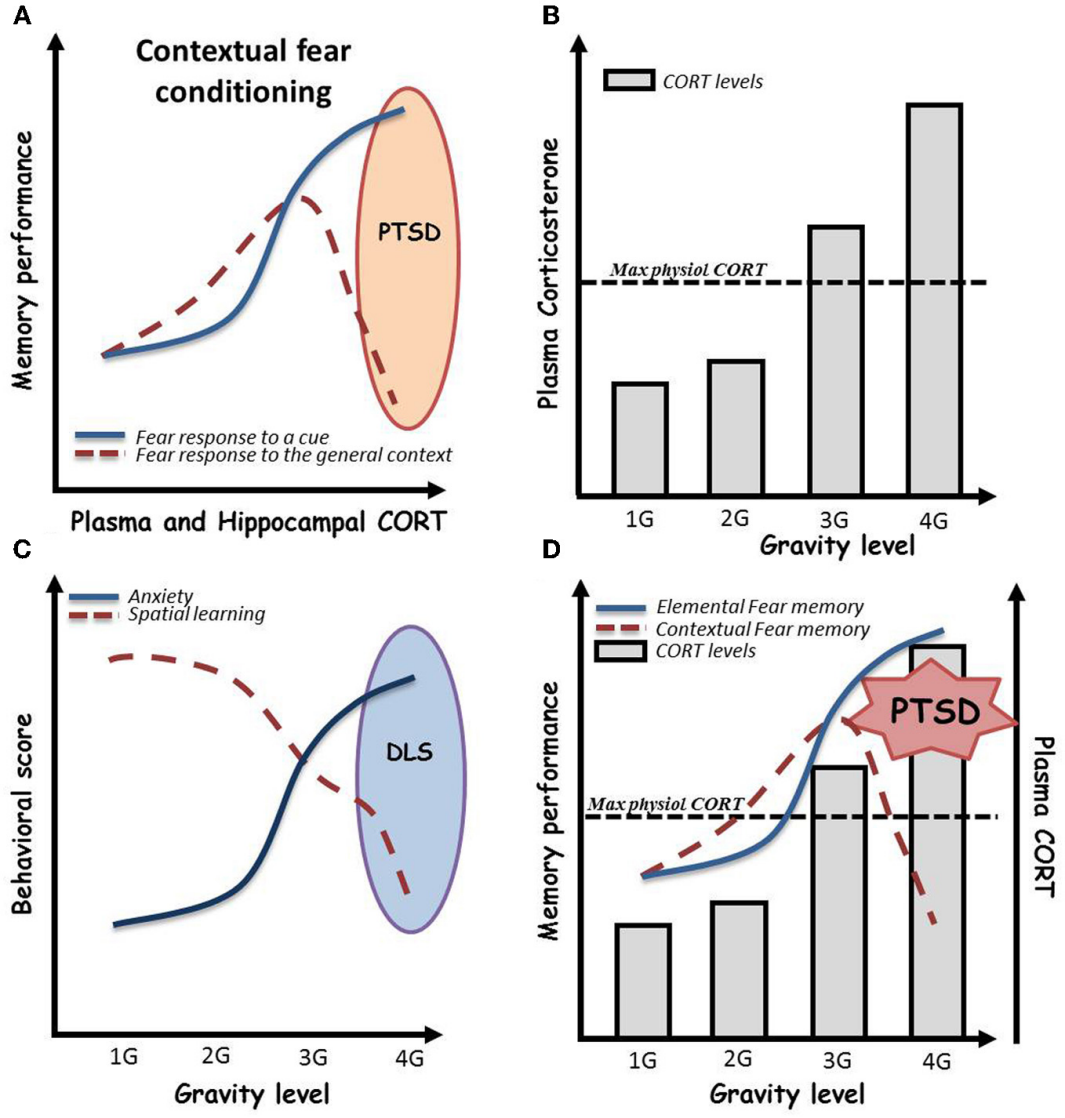
**(A)** Effects of acute stress, as assessed by plasma and hippocampal corticosterone (CORT) levels, on contextual *vs.* elemental fear memory. Globally, stress is associated with beneficial effects on both procedural cued and relational memories. However, in a given environment in which a negative event occurs, and under high emotional conditions, subjects can develop hypermnesia for a salient cue of the environment associated to amnesia for the general context of the event. This paradoxical pattern, well described from the psychological point of view, has been named “Post-traumatic stress disorder” (PTSD). As depicted here, recently, Kaouane and colleagues modeled a PTSD-like syndrome in mice by increasing systemic and hippocampal CORT levels after contextual Pavlovian conditioning, demonstrating the importance of stress hormone concentration in the trauma. **(B)** Effects of gravity level on plasma CORT in mice. Plasma CORT levels increase beyond the physiological maximal levels when mice are bred under more than 3 G conditions by centrifugation [adapted and schematized from Guéguinou et al. (2012)]. **(C)** Persistent effects of gravity level on anxiety and hippocampal learning in mice. Increasing levels of gravity (from 1 to 4 G) dramatically increase persistent (>10 days) anxiety troubles associated to spatial learning impairments in the Morris water maze. We then hypothesize a possible depressive-like syndrome in mice bred under high gravity level conditions. **(D)** Expected psycho-physiological pattern of mice bred under high gravity level conditions. High gravity level breeding conditions induce an increase in plasma and hippocampal CORT levels beyond the maximal physiological levels. As shown in this combined scheme, we hypothesized that these CORT levels could impair contextual conditioning during centrifugation. This could lead to a paradoxical pattern in which mice initially trained to associate a global environment with an electrical shock would display fear memory for a salient cue and amnesia for the context in its whole. In summary, 4 G centrifugation associated with a negative event could induce PTSD-like syndrome in mice.

It is well known that mood (anxiety, depression) and arousal (stress), that are very closely related, dramatically modulate the encoding and retrieval of memory (Yerkes and Dodson, 1908; Roozendaal, 2000; Lupien et al., 2007; Xu et al., 2011). Yerkes and Dodson initially described an inverted U curve where increasing arousal promotes encoding and relevant retention of memory until a limit after which it becomes deleterious. Since then, numerous data where published and the effect of stress on learning capacities has been yet extensively detailed. It is now quite accepted that anxiety and stress effects vary as a function of the type of memory considered. Strikingly, it seems that very high levels of stress tend to specifically alter relational memories (for example spatial, contextual) to the benefit of procedural ones (for example cued fear learning) (Packard, 2009), sometimes even leading to pathological profiles such as post-traumatic stress disorder (PTSD, Figure 2A) (Kaouane et al., 2012).

On the other hand, astronauts often experience mood disorders in space (Kanas, 1990, 1998; Carter et al., 2005), due to the conjunction of action of various physiological and psychosocial stressors, not always related to the decrease in gravity force level. Among those stressors can be cited the confinement, lack of activity, non-appetitive food, solar radiations, loss of light-dark cycle, or deregulation of circadian rhythms, that are unavoidable in space-flights, but cannot be tested in their multiplicity in ground-based studies. Indeed, the lack of physical activity and the redistribution of corporal fluids during long lasting head-down tilting bed-rest (Parker et al., 1983; Ishizaki et al., 1994, 2000, 2002; Styf et al., 2001), as well as confinement in space or in ground-based studies (Shimamiya et al., 2005) often result in the appearance of depressive-like behaviors and feelings. Thus, one of the major concerns of future investigation in the field of space biology will certainly be to explain the mechanisms underlying mood disorders in space, and try to correlate it with cognitive defects.

To this purpose, as sending humans and animals in space is technically and economically very hard to support, the first step have been to design models of gravity manipulations that are easily reproducible and applicable to behaving subjects. Concerning microgravity, three groups of models can be proposed proposed. The first model, parabolic flights, allows putting directly subjects into microgravity conditions but only during a few seconds, which can be used as acute stressor but is obviously not sufficient to induce mood abnormalities. The two others, would only mimic the physical secondary effects of the lack of gravity vector, either by decreasing external forces applied on the body (hypobaric box, only used in the context of cardiac deconditioning) (for review, see Foster and Butler, 2009); or by applying new forces leading to corporal fluids redistribution (hind-limb unloading by tail suspension in rodents) (Woodman et al., 1991, 1993; Henriksen et al., 1993; Tischler et al., 1993). In this context, a recent work showed that a two-week chronic tail suspension induces a loss of sucrose preference and lateral hypothalamus auto-stimulation in young Sprague–Dawley male rats (Moffitt et al., 2008). This anhedonia appeared to be conjugated with sympatho-vagal imbalance in cardio-vascular tone modulation, and such a stress induced imbalance have been shown to be sensitive to classical anti-depressors, namely selective inhibitors of serotonin recapture, like Fluoxetine (Grippo et al., 2006). Consistent with these results, microgravity and simulated weightlessness have been shown to impact in serotonergic system in the brain, especially leading to anorexia and hippocampal modulation defects (Horrigan et al., 1997; Blanc et al., 1998; Varma et al., 2000; Da Silva et al., 2002).

By contrast, effects of gravity on general physiology can be assessed by increasing the G force level imposed to the subjects. To this purpose, centrifuges able to house cages of rodents have been designed, allowing the breeding of animals under hypergravity conditions. In the context of mood and affect, it has been shown that mild acute centrifugation (2 G during 2 h) in peri-adolescent CD1 mice induced mild and sex-dependent anxiety as assessed by elevated plus maze test (Francia et al., 2004), whereas chronic (3 weeks) exposure of 2 month-old C57BL6j mice to 2 G or 3 G elicited highly anxiogenic profile in light-dark box associated with high levels of corticosterone in the blood serum (Figure 2B) (Guéguinou et al., 2012). Additionally, preliminary experiments in the Morris water maze, after chronic 3–4 G centrifugation, noted an increase of floating, a parameter that has been described as indicator of depressive-like symptoms in rodents (Figure 2C) (Stewart et al., 1996; Schulz et al., 2004, 2007a,b). Additionally, a series of experiments showed, thanks to Fos immunohistochemistry, that 90 min of 2 or 4 G centrifugation (Gustave Dit Duflo et al., 2000; Nakagawa et al., 2003), as well as return from a 2 week-long space flight (Pompeiano et al., 2001), induce activation of the amygdala in rats. Yet, this cerebral structure, through its relations with the hippocampus, has been implicated in treating highly emotional components of memorized episodes (Desmedt et al., 1998; Bianchin et al., 1999; Ferry et al., 1999; Frey et al., 2001; Akirav and Richter-Levin, 2002; Quevedo et al., 2003; Dolcos et al., 2004), including in extreme situations that lead to psychopathologies such as PTSD (Kaouane et al., 2012). Indeed, activation of the amygdala following application of decreased or increased gravity force may illustrate the fact that animals not only underwent physiological stress but really experienced emotional trauma. We thus hypothesized that high gravity level breeding conditions can induce an increase in plasma and hippocampal CORT concentrations beyond the maximal physiological. As a consequence depicted in Figure 2D, these CORT levels could modulate learning and, according to Kaouane et al. (2012), if associated to a negative event, impair contextual learning during centrifugation. This could lead to a paradoxical pattern in which, for instance, mice initially trained to associate a global environment with an electrical shock would display fear memory for a salient cue and amnesia for the context in its whole. In summary, centrifugation associated with a negative event could induce memory impairments such as PTSD-like syndrome in mice.

As a partial conclusion, one can argue that when a subject is submitted to non-terrestrial gravity conditions, he undergoes physiological adaptations that can evoke deregulations in mood, affect, and arousal systems, resulting in the development of despair, anxiety– and/or depressive-like behaviors. From this line of evidence, and knowing that mood and arousal are direct modulators of memory acquisition and retention, one can consider the importance of studying these functions under modified gravity conditions.

## Gravity changes and neuronal function

Memory is globally defined as the capacity of an organism to store pertinent information from its environment in order to use it adequately when re-exposed to the same situation. In animals, the capacity of acquiring data in a given situation relies on the *coordinated and reverberating* activity of sensorial systems (immediate memory, attention) that can lead to an increased sensitivity of neurons into the cerebral structures dedicated to the encoding of memory (short term memory) (Hebb, 1949). Then, when data are pertinent enough, the increased capacity of neurons to respond to the same stimuli can be maintained so as to permanently strengthen synapses activity. This is the so called Long Term Potentiation (LTP), a very conservative phenomenon that has been described in numerous species ranging from cephalopods (Shomrat et al., 2008) to non-human primates (Urban et al., 1996), and frequently presented as the substrate for memory traces (Teyler and Discenna, 1984; Eccles, 1986; Greenough, 1987; Miller and Mayford, 1999; Lynch et al., 2007). Indeed, as it is well known from the molecular and cellular point of views, and now relatively easy to evidence *ex vivo* by the way of electrophysiological devices, a first step to study potential effects of gravity on learning and memory would be to determine its effects on LTP. However, very few articles have been published up to now. For instance, unfortunately to our knowledge, no studies were conducted on the effects of prolonged weightlessness or simulated weightlessness on LTP. However, some works have yet been interested in the “hypergravity side of the force.” First, it has been reported that LTP was preserved in the rat hippocampus (one of the main structures implicated in memory encoding and storage) after either 2 days or 14 days of mild (2 G) centrifugation (Guinan et al., 1998). This, as we will next see, is consistent with the fact that individuals submitted to mild hypergravity forces are still able to learn tasks, even if mild impairments can be noted. More recently, Ishii and collaborators even showed that sub-chronic (48 h) centrifugation (4 G) stress is sufficient to induce LTP in the hippocampus. As no or few mechano-receptors have yet been described in this structure to eventually physically detect gravitational chances, authors proposed that LTP induction could be secondary to the action of stress hormones (Ishii et al., 2004). However, the possibility that hypergravity may be beneficial for LTP stays still very controversial. Indeed, the development of a synaptopathy characterized by incomplete filling of glutamate vesicles and reduced exocytosis competence has been recently reported in brain synaptosomes of rats submitted to acute (1 h) 10 G centrifugation (Krisanova et al., 2009).

In cells, the molecular nature of gravity sensor is not yet characterized; it could be a component of the primary cilium because its function is the detection of movement and the polarity of the cell (Moorman and Shorr, 2008). But the TRP like channels named PIEZO will be candidate to decode the variation of pressure and gravity at the cellular level because these channels can be activated by stretch and pressure changes on the plasma membrane (Coste et al., 2010, 2012). This raises the possibility that physiological effects of gravity on synaptic transmission, and more generally on cerebral functioning, might closely depend on the level and the duration of application, what makes very difficult the choice of pertinent centrifugation protocols. Should it be more interesting to simulate acute (space-shuttles launch/landing) or chronic (life in space or on other planets) effects of gravity changes? In the first case, the very short duration of hypergravity application might be insufficient to induce observable effects and contrarily the very high level of gravity used can pollute eventual results by pushing individuals under extremely stressing conditions. On the other hand, trying to coordinate the ground-based experiments on animal models with long duration flights of astronauts brings to the question of non-equivalent life duration and biological development across species. Finally, it is important to keep in mind that all the papers presented herein rely on experiments done a posteriori, i.e., under normo-gravity conditions *after* exposition to G-force changes.

From the molecular point of view, LTP requires transcription and translation of numerous genes leading to the production of new proteins necessary for the rearrangement of synapses (number of receptors, ion channels, structural proteins, growth factors, etc.) and stabilization of newly acquired memories (for reviews, see Bailey et al., 2004; Alberini et al., 2006). Up to now, two studies were interested in determining the changes in gene expression in the brain under modified gravity conditions. First, Frigeri et al. (2008), put in evidence, in C57BL6j mouse brain after 2 weeks of caudal suspension, an up-regulation of the gene encoding for NMDAR1, a fundamental subunit of NMDA receptors which activation is at the origin of the induction of LTP (Frigeri et al., 2008). Oppositely, Del Signore and co-workers showed that five daily repeated 1 h 2 G centrifugations increased hippocampal expression of *Syndet* gene which encodes for an SNAP-25 related protein probably involved in changes in synaptic transmission. Interestingly, authors also showed substantially elevated expression of proSAAS, a peptide closely related to stress response and control of pain (Del Signore et al., 2004).

Even if comparative analysis of these recent works points as evident the possibility that gravity cognitive effects could have molecular explanations in terms of stress modulation of LTP, it is regrettable to note that data remain still poor. Moreover, it seems quite difficult to attest whether these molecular arguments are sufficient to attest for cognitive effects. In this context, it should be interesting to perform a transcriptomic study to describe the entire C57BL6j mouse hippocampal genes expression under chronic (21 days) hypergravity conditions, as an alternative to find the molecular targets affected by hypergravity and implicated in neuronal and/or glia cell activity during learning and memory processes.

## Gravity changes and learning

Considering the cognitive point of view, even if a great amount of studies pointed effects on sensori-motor skills and procedural learning under microgravity (in space- or parabolic-flights) or after centrifugation, the literature is very poor about the effect of gravity on more superior functions. Indeed, one of the most exciting capabilities of the brain is to be able to consciously encode declarative data such as biographical episodes (episodic memory) or general knowledge about the environment (semantic memory), and recall it later to adapt the behavior to a similar situation (Squire, 2004). In this context, one of the best studied fields of investigation is the spatial memory, which depends on the integrity of the hippocampus as a structure able to build and store mental representations of the environment (O'Keefe and Nadel, 1978; Redish, 1999). On earth, graviperception and navigation mostly rely on detection of the body position and movements (Potegal, 1982; Strelow, 1985; Rieser, 1990; Loomis et al., 1993). This is achieved by the vestibular machinery which, in conjunction with the visual system, permits the detection of vertical, horizontal, and angular accelerations, as well as the perception of longitudinal body axis. Yet, vestibular influences on CA1 hippocampal neurons have been shown in rats *in vivo* (Horii et al., 2004). Specifically, it appears that inactivation, or lesion, of the vestibular system in rats, transitorily abolish the firing specificity of place and head direction cells in the hippocampus (Stackman et al., 2002; Russell et al., 2003b). Concordant with these data, vestibulo-lesionned rats (Stackman and Herbert, 2002) and humans (Peruch et al., 1999) experience difficulties when trying to navigate in the absence of visual landmarks, and are impaired in achieving a classical spatial radial arm maze task (Russell et al., 2003a).

Thus, accumulating evidence suggests that navigation, spatial learning, and by extension declarative memory, can be modulated by gravity environment. Unfortunately, when analyzing microgravity literature, a first comment would be that very few studies are available in this field of investigation, and they are not always in accordance the ones with the others. Indeed, the perceptions of orientation (Dyde et al., 2009) and longitudinal body axis (Jenkin et al., 2003, 2005, 2011; Clement et al., 2007) are clearly altered during microgravity periods in parabolic flights, even if well trained astronauts seem not to be affected by weightlessness when tested in mental rotation of three dimensional objects (Leone et al., 1995). In rats, it has been shown that place cells develop three dimensional firing specificity in space, while place fields are only two dimensional on earth (Knierim et al., 2000). Yet, spatial learning and memory as assessed in a Morris water maze task, seem to be preserved after early development in microgravity (Temple et al., 2002).

When considering hypergravity side, results are yet much more unclear. Two studies in rats found that very high levels of gravity (10–15 G during 3–5 min) induce neuronal hippocampal apoptosis and spatial learning and memory impairment in Y and Morris water mazes (Sun et al., 2009; Feng et al., 2010). Although interesting, these results do not really account for hypergravity effects on memory. It is more reasonable to attribute them to intense acute physical and psychological stress. Indeed, when bringing so rapidly any organism to such a high level of gravity by centrifugation, we cannot rule out the potential roles of sensorial perturbations, corticotropic axis runaway, cardio-vascular decompensation or apoptosis of other cellular types than neurons, in generating cognitive impairments.

Contrarily, several studies used much milder centrifugation protocols ranging from 2 to 4 G, either acutely (1–2 h) or chronically (3–21 days), to more specifically target the part of gravity level variation in disrupting learning and memory. Acute (2 h) or repeated acute (1 h per day during 5 days) 2 G centrifugation alters the discrimination of new spatial arrangements (Mandillo et al., 2003) as well as initial acquisition of spatial Morris water maze task. However, the same procedure is beneficial on flexibility as assessed by water maze reversal test in adolescent (Francia et al., 2004) or adult CD1 mice treated during infancy (Francia et al., 2006). In the same order of idea, after two weeks of 2 G centrifugation, only the initial phase of radial arm maze acquisition seems to be affected, this delayed acquisition being rapidly compensated by hypergravity induced hyperactivity (Mitani et al., 2004). This last result can be retained as the most relevant one as it takes into account a possible adaptation of the physiology to the hypergravity condition stress.

## Conclusion and perspectives

In conclusion, it appears that hypergravity effects on learning and memory vary as a function of the duration of centrifugation, the level of gravity imposed to the subjects, and the behavioral paradigm in which they are tested. However, independently of the protocols' discordance, one can note that for obvious reasons, all studies refer to *a posteriori* effects of hypergravity on cognition. It becomes then evident that a large contribution should be brought to our knowledge by identifying *on line* effects of hypergravity on memory encoding and more generally animal behavior. That is why we think necessary to develop a totally automated apparatus that could be placed in the centrifuge, and in which various form of memories (ranging from declarative to non-declarative ones), as well as anxiety, could be tested. Such a device will allow dissociate the weights of mood, anxiety, stress, and gravity force on cognition, by varying levels and durations of centrifugation and by using pharmacological treatments. Moreover, it could be useful to annihilate all experimental bias commonly encountered in behavioral experiments. This might alleviate stress of training and testing, and animals would have the opportunity to virtually choose the moment in which they enter the modules containing behavioral tests, and follow to live in social colony conditions during all the procedure of centrifugation.

### Conflict of interest statement

The authors declare that the research was conducted in the absence of any commercial or financial relationships that could be construed as a potential conflict of interest.
